# Taking 200 mg Vitamin C Three Times per Day Improved Extraction Socket Wound Healing Parameters: A Randomized Clinical Trial

**DOI:** 10.1155/2022/6437200

**Published:** 2022-03-10

**Authors:** Nanthanut Pisalsitsakul, Chaiwat Pinnoi, Narueporn Sutanthavibul, Paksinee Kamolratanakul

**Affiliations:** ^1^Department of Oral and Maxillofacial Surgery, Faculty of Dentistry, Chulalongkorn University, Bangkok 10330, Thailand; ^2^Department of Pharmaceutics and Industrial Pharmacy, Faculty of Pharmaceutical Sciences, Chulalongkorn University, Bangkok 10330, Thailand

## Abstract

Vitamin C is essential for wound healing. However, there are no reports concerning the effect of a different dose of vitamin C on extraction wound size clinically. Therefore, the aim of this study was to investigate the effect of different oral vitamin C doses on extraction wound healing. A split-mouth, double-blind randomized clinical trial was performed in 42 patients who underwent symmetric bilateral noninfected premolar extraction. The patients were randomly divided into 3 groups, namely, P/600, P/1,500, and 600/1,500 (14 patients for each group); P/600: placebo vs. 600 mg vitamin C/d, P/1,500: placebo vs. 1,500 mg vitamin C/d, and 600/1,500: 600 mg vitamin C/d vs. 1,500 mg vitamin C/d. Patients were prescribed placebo or/and vitamin C three times a day for 10 days after each tooth extraction. Extraction wound size and pain score were evaluated. The wound assessment was performed on day 0, 7, and 21; and then the tooth on the other side was extracted using the same protocol. Pain score was recorded on the first three days after extraction. The reduced size of mesiodistal extraction wound in percentage reduction between day 0 and 7 of teeth receiving vitamin C 600 mg/d was more than that in placebo (*P* < 0.05). Pain scores on day 1–3 of teeth receiving vitamin C 600 mg/d were significantly lower than the placebo side (*P* < 0.05). Taking oral vitamin C 600 mg/d over three doses for 10 days after tooth extraction enhances extraction wound healing by reducing mesiodistal extraction wound and reduces postoperative pain.

## 1. Introduction

Wound healing has been studied for decades, however, the wound healing process after tooth extraction is still not fully understood. Although tooth extraction is a common dental procedure, there are common minor postoperative complications, such as delayed wound healing that impacts patients' quality of life and increases clinical workload and cost [1].

The wound healing process requires both macronutrients and micronutrients [2, 3]. Delayed wound healing is a failure to progress through the normal phases and nutritional deficiencies, such as vitamin C that humans have to obtain from their diet, which is a significant factor in all phases of wound healing [3]. In the inflammatory phase, vitamin C is required for neutrophil apoptosis and clearance. In the proliferative phase, vitamin C is involved in collagen synthesis, maturation, secretion, and degradation by acting as a cofactor for proline and lysine hydroxylation during collagen synthesis and is associated with fibroblast proliferation, which affects angiogenesis and capillary strength [2–4].

The role of vitamin C in wound healing has been investigated in laboratory and animal studies [5–8]. However, there are few clinical studies in humans, especially concerning oral wound healing [9–11]. Two studies reported that 500 mg vitamin C taken orally 3 or 4 times/day (1,500 mg/d and 2,000 mg/d) promoted extraction wound healing [10, 11]. In contrast, our in vitro study found that rinsing with a low dose (20 *μ*g/ml) of vitamin C induced better fibroblast migration, proliferation, and gingival wound closure compared with a higher dose (50 *μ*g/ml) of vitamin C. Moreover, the higher dose of vitamin C reduced cell viability in oral fibroblast culture [12]. The absorption of oral vitamin C was highest at a dose of 200 mg [13]. Based on the bioavailability of vitamin C [13], we hypothesized that a lower dose might promote wound healing. Currently, there are no reports concerning the effect of a lower dose (200 mg) of vitamin C 3 times/day or the effect of vitamin C on extraction wound size. Therefore, the purpose of this split-mouth clinical study was to investigate the efficacy and to compare two different oral doses (600 mg/d and 1,500 mg/d) of vitamin C on extraction wound healing based on wound size as the primary outcome. Moreover, we evaluated pain score as a secondary outcome.

## 2. Materials and Methods

### 2.1. Patient Acquisition

We performed a split-mouth, double-blind, randomized controlled clinical trial at the Oral and Maxillofacial Surgery Clinic at the Faculty of Dentistry, Chulalongkorn University, from August 2018 to August 2019. The study protocol was approved by the Human Research Ethics Committee of the Faculty of Dentistry, Chulalongkorn University (HREC-DCU 2018–031) and registered with the Thai Clinical Trial Registry (ID #TCTR20180830002). G*∗*Power version 3.1 software was used for the sample size calculation. Based on our pilot study, the calculated sample size was 8 patients per group. Drop-out compensation was calculated and the sample size was increased by 40% per group. Therefore, the total sample size of three groups was 42 patients (14 patients per group), with 168 extraction sites.

The patients were informed about the advantages and disadvantages of the study and provided informed consent before enrollment. The inclusion criteria were healthy patients 14–40 years old [14–16] with no medical conditions with symmetric bilateral noninfected premolars requiring extraction for orthodontic treatment without tooth pathology, bone loss, or defect. The exclusion criteria were as follows: smokers, alcoholics, pregnant or lactating women, psychiatric patients, patients taking corticosteroids or estrogen-containing contraceptive drugs, and patients who missed more than 1 dose of vitamin C.

The enrolled patients were randomly assigned using block randomization into 3 different paired-groups (*n* = 14); placebo vs. 600 mg vitamin C/d (P/600), placebo vs. 1,500 mg vitamin C/d (P/1,500), and 600 mg vitamin C/d vs. 1,500 mg vitamin C/d (600/1,500).

### 2.2. Surgical Protocol

At the first visit, each patient had the experimental site randomly selected by one research examiner. Their vital signs were recorded before the extraction. The operations were performed by the same surgeon using a standardized technique, using elevators and adapted forceps under local anesthesia consisting of 2% mepivacaine with 1 : 100,000 epinephrine. After tooth extraction, bleeding was stopped using gauze and pressure. Every patient was given the same postoperative instructions. An analgesic drug, 500 mg acetaminophen, was prescribed as needed. The tooth on the other side of the mouth was extracted with the same method 21 days after the first extraction.

There were two different interventions given to each patient after tooth extraction on each side. The patients were blinded to the intervention they were prescribed after each extraction.

The patients were prescribed to take placebo pills or vitamin C pills (600 mg or 1500 mg daily) orally for three times daily after meals for 10 days after extraction. The vitamin C used in the study was from Patar Lab Co. (Pathum Thani, Thailand). However, the placebo calcium carbonate, sucrose, and carnauba wax coating were manufactured under the license of the Faculty of Pharmaceutical Sciences, Chulalongkorn University.

### 2.3. Data Collection

**Figure 1:**
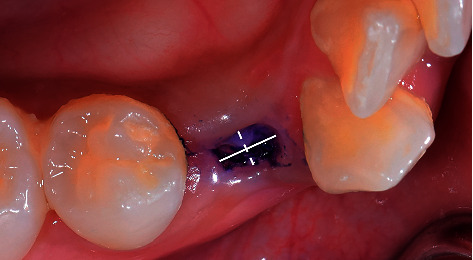
Measurement of wound socket in bucco-lingual and mesio-distal dimension using caliper. MD (thick line), mesio-distal measurement; BL (dashed line), bucco-lingual measurement.

**Figure 2:**
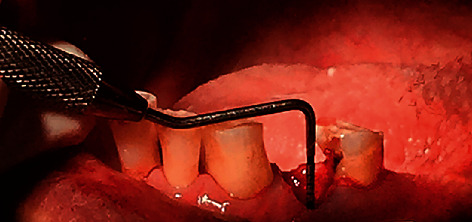
Measurement of the wound socket depth using a periodontal probe. The reference point was measured at the middle of the buccal gingiva between the adjacent teeth.

Each extraction wound site was measured 3 times (immediately after tooth extraction as a baseline and on day 7 and day 21 after extraction) by two blinded examiners who were experienced oral surgeons. The inter-rater reliability of the two examiners who evaluated the wound healing parameters was determined by the intraclass correlation coefficient (ICC). The data were compared and the reliability test demonstrated an intraclass correlation coefficient of 0.771, which indicated good reliability [13]. The measurement procedures were as follows:

- The extraction socket size was measured (rounded up to the nearest 0.5 mm) with a caliper in the bucco-lingual (BL) and mesio-distal (MD) dimension using the cementoenamel junction level of the adjacent teeth as the reference level. The healing socket area was identified using toluidine blue staining (Figure 1).
- The extraction wound depth (rounded up to the nearest mm) was measured at the middle of the buccal plate between the adjacent teeth using a periodontal probe (PCPUNC15, Hu-Friedy) (Figure 2).

The patients were asked to list their meals on a form for 7 days after extraction to determine the amount of vitamin C in their diet. Moreover, the pain score using a 10-point visual analog scale from 0 (no pain) to 10 (extreme pain) was recorded by each patient on the first three days after extraction at 8 am, 12 pm, and 6 pm. Every patient was instructed to record their pain score before taking an analgesic drug.

### 2.4. Statistical Analysis

All data analyses were performed using the statistical program SPSS version 22. The patients' demographic data are presented as descriptive statistics. The normal distribution of the data was tested using the Shapiro–Wilk's test. The reduced size of the extraction wound at each time point was calculated as follows:

(1) $$\begin{array}{c} \text{Measurement}=\frac{{\text{Measurement}}_{\left(\text{immediate}/\text{day }7\text{ after extraction}\right)}-{\text{Measurement}}_{\left(\text{day }7/\text{day }21\text{ after extraction}\right)}}{{\text{Measurement}}_{\left(\text{immediate}/\text{day }7\text{ after extraction}\right)}}. \end{array}$$

All proportions were calculated as a percentage. Because of the nonnormal distribution, the reduced wound size proportion as a percentage of the extraction wound between the 2 extraction sites and pain scores after the 2 extractions were compared using the Wilcoxon signed-rank test. Differences were considered significant at a *P* value <0.05.

## 3. Results

Thirty-two patients (128 extraction sites) were included in this study (10 drop-out patients). There were 12 patients (24 extraction sites for placebo and 24 extraction sites for vitamin C600 mg) in the P/600 group and 10 patients (20 extraction sites for placebo and 20 extraction sites for vitamin C600 mg) in the P/1,500 and 600/1,500 groups. The patients comprised 13 males and 19 females. The mean age of the patients was 18.68 ± 3.95 years, range 14–28 years. The extraction sites were mostly in the maxilla (57.1%). No complication was found during the healing period.

Comparing the percentage reduction in the extraction wound size between the extraction sites, the P/600 group demonstrated a significantly greater reduction in the MD wound size compared with the placebo group (57.3% vs. 48.3%) between day 0 and day 7 (*P*=0.036) (Table 1). However, the wound size reduction between the extraction sites in the P/1,500 (Table 2) and 600/1,500 groups (Table 3) were not significantly different (*P* > 0.05).

Moreover, the mean VAS scores on day 1–3 for patients receiving vitamin C were significantly lower than placebo in the P/600 group (*P* < 0.05). There was no significant difference in the mean VAS scores on day 1–3 between extraction sites in the other two groups (Table 4).

## 4. Discussion

The present study was a double-blind, randomized, controlled clinical trial with a split-mouth design to remove confounding factors from intersubject variability [17]. We evaluated the dimensions, BL, MD, and socket depth, of extraction wound healing related to vitamin C supplementation and wound size measurement was performed to provide reliable objective data.

The definitive vitamin C regimen is currently unresolved, and there are no specific guidelines for prescribing vitamin C for wound healing [18]. Our previous in vitro study revealed that a lower dose of vitamin C promoted fibroblast migration and in vitro wound closure [12]. Moreover, the absorption of oral vitamin C was 100% at a dose of 200 mg, the absorption at doses of 500 mg and 1,250 mg were <75% and <50%, respectively [13]. Therefore, increasing the dose of oral vitamin C results in less efficient absorption due to transporter saturation. Vitamin C is transported through the intestinal epithelium via sodium-ascorbate cotransporters (SVCTs) and SVCT1 [19]. Melethil et al. [20] reported that the gastrointestinal absorption and renal tubular reabsorption of vitamin C were dose-dependent processes and renal excretion occurs only when the plasma vitamin concentration exceeds a threshold value. It was recommended to prescribe vitamin C in divided doses over the course of a day to maximize vitamin C uptake and plasma concentration [21]. In the hypermetabolic state, the recommended dosage was between 500 mg and 2,000 mg per day, which was more than 10-fold the recommended daily intake suggested by the Food Standards Agency [22]. The recommended dose of vitamin C to promote healing was 500–1,000 mg per day [4]. Altogether, we hypothesized that dividing the total dose of 600 mg per day into 200 mg three times daily might be more effective in promoting wound healing based on the bioavailability of vitamin C.

In the P/600 group, the significant percentage reduction of the MD extraction wound size on the 600 mg/d vitamin C side on day 7 (*P* < 0.05) was possibly due to fibroblast differentiation. The blood clot in an extraction wound is replaced by granulation tissue in 1 week and granulation tissue formation and collagen deposition occur from day 4–14 after injury. Wound contraction begins after the granulation tissue is well-established [23]. The lower dose of vitamin C (600 mg/d) enhanced the healing process and resulted in faster wound closure. In contrast, the higher dose of vitamin C (1,500 mg/d) resulted in similar extraction wound healing compared with the lower dose of vitamin C (600 mg/d) (*P* > 0.05). Vitamin C possibly enhanced wound healing at both 600 mg/d and 1,500 mg/d, however, there were no significant differences between the doses in the 600/1,500 group. This may be because at both 600 and 1,500 mg vitamin C the uptake is already saturated, which might result in no clinical difference between the groups.

Bone loss and collapse of the surrounding gingiva occur in the normal healing response to tooth extraction. The bone resorption occurs in the bucco-lingual and apicocoronal dimensions [24]. However, the mesio-distal dimension is supported by interdental bone and intact adjacent teeth. Therefore, we assumed that the mesio-distal change in the extraction wound size was possibly due to gingival tissue healing without the related effect of bone socket collapse. How vitamin C affecting bone socket healing may need further investigation. Although the analgesic mechanism of vitamin C is unclear, a previous study reported a correlation between vitamin C and pain control [25]. Li et al. [26] found that 300 mg/d vitamin C did not decrease postoperative pain in dental implant surgery; however, the present study found that 600 mg/d vitamin C reduced postoperative pain after tooth extraction. Mohammed et al. [7] also reported that vitamin C accelerated the termination of the inflammatory phase. Pain is a symptom of inflammation, thus these results imply that the shorter the inflammatory phase, the less the pain occurs. Because vitamin C is an antioxidant, it can reduce cell damage and reduce inflammation by decreasing inflammation markers, such as C-reactive protein and proinflammatory cytokines [25].

The baseline vitamin C levels in each patient are a limitation of this study. However, we controlled the individual variation between patients by using a split-mouth study design and comparing each individual extraction site with the % reduction of the extraction wound each site. The amount of vitamin C-containing diet was approximately 10-fold lower than our experiment doses that we prepared in this study. The vitamin C-containing diet may not strongly affect the healing outcome. Tight controls for a high vitamin C-containing diet are recommended for future study.

## 5. Conclusions

In conclusion, this study demonstrated that 600 mg/d (200 mg three times daily) oral vitamin C promoted tooth extraction wound healing in the mesio-distal dimension and reduced postoperative pain. Early tooth extraction wound healing might promote earlier placement of an implant, which would be beneficial to the patient. A larger population with other dental procedures, including patients before preprosthetic treatment and dental implantation, immunocompromised patients, and patients with a risk of delayed wound healing, are recommended.

## Figures and Tables

**Table 1:** % reduction in mean wound size between the 2 extraction sites in the P/600 group (placebo vs. 600 mg vitamin C/d).

	% reduction of the extraction wound at the placebo side	% reduction of the extraction wound at the 600 mg vitamin C/d side	*P* value^a^
*Immediate and 7 days*			
Bucco-lingual	45.9	47.5	0.575
Mesio-distal	48.3	57.3	0.036^*∗*^
Depth	61.1	68.2	0.122

*Immediate and 21 days*			
Bucco-lingual	54.8	58.9	0.445
Mesio-distal	54.6	60.2	0.262
Depth	83.3	86.2	0.398

*7 days and 21 days*			
Bucco-lingual	13.8	18.7	0.575
Mesio-distal	11.3	5.9	0.416
Depth	56.8	49.6	0.540

SD, standard deviation. ^a^*P* value, Wilcoxon signed-rank test. ^*∗*^*P* < 0.05.

**Table 2:** % reduction in mean wound size between the 2 extraction sites in the P/1,500 group (placebo vs. 1,500 mg vitamin C/d).

	% reduction of the extraction wound at the placebo side	% reduction of the extraction wound at the 1,500 mg vitamin C/d side	*P* value^a^
*Immediate and 7 days*			
Bucco-lingual	52.2	47.5	0.233
Mesio-distal	37.0	36.5	0.953
Depth	47.1	60.2	0.066

*Immediate and 21 days*			
Bucco-lingual	59.2	58.4	0.575
Mesio-distal	49.4	48.9	0.674
Depth	82.3	88.1	0.069

*7 days and 21 days*			
Bucco-lingual	12.6	20.0	0.225
Mesio-distal	16.6	16.8	0.917
Depth	66.7	67.2	0.953

SD, standard deviation. ^a^*P* value, Wilcoxon signed-rank test. ^*∗*^*P* < 0.05.

**Table 3:** % reduction in mean wound size between the 2 extraction sites in the 600/1,500 group (600 mg vitamin C/d vs. 1,500 mg vitamin C/d).

Mean difference	% reduction of the extraction wound at the 600 mg vitamin C/d side	% reduction of the extraction wound at the 1,500 mg vitamin C/d side	*P* value^a^
*Immediate and 7 days*			
Bucco-lingual	48.2	42.3	0.767
Mesio-distal	45.0	44.6	0.953
Depth	69.4	70.0	0.753

*Immediate and 21 days*			
Bucco-lingual	58.1	56.4	0.953
Mesio-distal	52.7	53.0	0.674
Depth	86.6	83.0	0.237

*7 days and 21 days*			
Bucco-lingual	20.2	22.0	0.674
Mesio-distal	14.1	13.8	1.000
Depth	53.0	41.1	0.160

SD, standard deviation. ^a^*P* value, Wilcoxon signed-rank test. ^*∗*^*P* < 0.05.

**Table 4:** Pain scores assessed by VAS (in mm).

Group	Time	Placebo	Vitamin C 600 mg/d	*P* value^a^
P/600 group (*n* = 10)	Postoperative day 1 (mean ± SD)	4.012 ± 2.71	1.664 ± 1.34	0.021^*∗*^
	Postoperative day 2 (mean ± SD)	2.725 ± 2.40	1.126 ± 1.25	0.045^*∗*^
	Postoperative day 3 (mean ± SD)	1.116 ± 1.33	0.582 ± 0.76	0.017^*∗*^
Group	Time	Placebo	Vitamin C 1,500 mg/d	*P* value^a^
P/1,500 group (*n* = 9)	Postoperative day 1 (mean ± SD)	2.001 ± 3.26	1.033 ± 2.05	0.063
	Postoperative day 2 (mean ± SD)	1.550 ± 3.07	0.327 ± 0.65	0.157
	Postoperative day 3 (mean ± SD)	1.326 ± 2.63	0.017 ± 0.34	0.157
Group	Time	Vitamin C 600 mg/d	Vitamin C 1,500 mg/d	*P* value^a^
600/1,500 group (*n* = 9)	Postoperative day 1 (mean ± SD)	1.622 ± 1.78	1.799 ± 1.15	0.608
	Postoperative day 2 (mean ± SD)	1.019 ± 1.58	1.088 ± 1.54	0.458
	Postoperative day 3 (mean ± SD)	0.776 ± 2.21	0.395 ± 0.69	0.892

VAS, visual analog scale. ^a^*P* value, Wilcoxon signed-rank test. ^*∗*^*P* < 0.05.

## Data Availability

The supporting data are included within the article.
